# Hyperglycemia in severe traumatic brain injury patients and its association with thirty-day mortality: a prospective observational cohort study in Uganda

**DOI:** 10.7717/peerj.10589

**Published:** 2021-01-15

**Authors:** Paul Matovu, Musa Kirya, Moses Galukande, Joel Kiryabwire, John Mukisa, William Ocen, Michael Lowery Wilson, Anne Abio, Herman Lule

**Affiliations:** 1 Department of General Surgery, School of Medicine, College of Health Sciences, Makerere University, Kampala, Uganda; 2 Department of Neurosurgery, Mulago National Referral and Teaching Hospital, Kampala, Uganda; 3 Clinical Epidemiology Unit, Uganda-Case Western Reserve University Research Collaboration, Kampala, Uganda; 4 Department of Surgery, Mulago Hospital Kampala, Kampala, Uganda; 5 Department of Surgery, Lira University, Lira, Uganda; 6 Heidelberg Institute of Global Health, Ruprecht-Karls-Universität Heidelberg, Heidelberg, Baden-Wuerttemberg, Germany; 7 Turku Brain Injury Centre, Division of Clinical Neural Sciences, Turku University Hospital and University of Turku,, Injury Epidemiology and Prevention Research Group, Turku, Finland; 8 Department of Surgery, Kampala International University Western Campus, Directorate of Research and Innovations, Kampala, Uganda

**Keywords:** Traumatic-brain-injury, Hyperglycemia, Prevalence, Mortality, Uganda

## Abstract

**Background:**

Traumatic brain injury (TBI) is a growing public health concern that can be complicated with an acute stress response. This response may be assessed by monitoring blood glucose levels but this is not routine in remote settings. There is a paucity of data on the prevalence of hyperglycemia and variables associated with mortality after severe TBI in Uganda.

**Objective:**

We aimed to determine the prevalence of hyperglycemia in patients with severe TBI and variables associated with 30-day mortality at Mulago National Referral Hospital in Uganda.

**Methods:**

We consecutively enrolled a cohort 99 patients patients with severe TBI. Serum glucose levels were measured at admission and after 24 h. Other study variables included: mechanism of injury, CT findings, location and size of hematoma, and socio-demographics. The main outcome was mortality after 30 days of management and this was compared in patients with hyperglycemia more than 11.1 mmol/L to those without.

**Results:**

Most patients (92.9%) were male aged 18–30 years (47%). Road Traffic Collisions were the most common cause of severe TBI (64.7%) followed by assault (17.1%) and falls (8.1%). Nearly one in six patients were admitted with hyperglycemia more than 11.1 mmol/L. The mortality rate in severe TBI patients with hyperglycemia was 68.8% (OR 1.47; 95% CI [0.236–9.153]; *P* = 0.063) against 43.7% in those without hyperglycemia. The presence of hypothermia (OR 10.17; 95% CI [1.574–65.669]; *P* = 0.015) and convulsions (OR 5.64; 95% CI [1.541–19.554]; *P* = 0.009) were significant predictors of mortality.

**Conclusion:**

Hypothermia and convulsions at admission were major predictors of mortality in severe TBI. Early hyperglycemia following severe TBI appears to occur with a tendency towards high mortality. These findings justify routine glucose monitoring and could form the basis for establishing a blood sugar control protocol for such patients in remote settings.

## Introduction

Traumatic brain injury (TBI) is a growing public health concern, topping injury-related deaths and disability (James et al., 2020). Globally, TBIs are estimated to cause over 8.1 million years lived with disability and mortality in 3% annually; burdening economies with significant health costs from treatment and prolonged neuropsychological sequelae (James et al., 2019). Over 90% of TBI related deaths disproportionately occur in low- and middle-income countries such as those in the African region (Johnson & Griswold, 2017). In Uganda, such deaths from TBI mainly result from fatal road traffic crashes (Zia et al., 2019) and delayed presentation to health facilities (Vaca et al., 2019). Described as a silent epidemic, the World Health Organization (WHO) projected that by 2020, road traffic injuries (RTI) will be a major cause of TBI and will rank 3rd as a leading cause of disability adjusted life years lost (World Health Organization, 2015). While the mortality from road traffic injuries appeared to decline between 1990 and 2017, there was a global increase in the incidence rates of traumatic brain injuries by 3.6%, with foreseeable irreversible health consequences (James et al., 2019).

In low-income country settings, severe TBI cases may not reach hospitals, which increases the likelihood of under-reporting (Ssebakumba et al., 2020). In Uganda, head injuries are one of the top four most common admission diagnoses, contributing to 45.3% of patients admitted to intensive care units and 75% of the injury specific mortality rate (Hsia et al., 2010). TBI was associated with the highest mortality rate of 40.1% in the intensive care unit at Mulago, one of Uganda’s National Referral Hospitals (Kwizera, Dünser & Nakibuuka, 2012).

Traumatic brain injuries are associated with an acute stress response mediated by the sympatho-adreno-medullary axis (Shi et al., 2016). This is marked by elevation in plasma cortisol, glucagon, insulin and catecholamines, glucose, lactate, none-sterified fatty acids (Bosarge et al., 2015). Blood glucose can thus be a bio-marker of this response. Hyperglycemia can worsen the underlying brain damage by causing free radical injury, apoptosis and tissue lactic acidosis (Galgano et al., 2017). Whereas efforts are made to prevent and manage the primary brain injury, secondary brain injury is often overlooked culminating in a “second hit phenomenon” (Galgano et al., 2017).

A number of studies have shown significant association between hyperglycemia, TBI severity and mortality (Kafakimd et al., 2016; Bosarge et al., 2015; Salim et al., 2009). In a prospective cohort involving 834 patients with severe TBI, it was found that persistent hyperglycemia (defined as average daily blood glucose levels greater than 149 mg/dl for the first week after injury), was associated with a four-fold increase in mortality (Salim et al., 2009). According to Walia & Sutcliffe (2002), hyperglycemia was more strongly predictive of the outcome of 338 patients with head injury compared to mean arterial blood pressures. However, there is a paucity of published literature on the African population, establishing the prevalence and association between serum glycemic levels and TBI mortality.

A study by Adeolu et al. (2015) had equivocal findings that contrast sharply with most studies done elsewhere (Salim et al., 2009; Walia & Sutcliffe, 2002). Importantly, as treatments for hyperglycemia become available, blood glucose control is attracting significant attention as a promising intervention for reducing the complications of TBI (Shi et al., 2016); although the optimal glycemic ranges for these patients have not yet been determined (Shi et al., 2016). However, the occurrence of hyperglycemia in TBI and its association with the patient outcomes has not been largely studied in our resource constrained setting since glucose monitoring is not a routine for these patients. Whilst prevention of primary TBI is key, stepping up post-crash care such as glycemic monitoring and control of modifiable factors that influence outcome for the injured, could reduce fatalities. This study thus aimed at determining the prevalence of hyperglycemia in severe TBI and clinical-demographic variables associated with 30-day mortality.

## Materials and Methods

### Study design

This was a prospective observational cohort study in which all patients with severe TBI were consecutively recruited for a period of 3 months and followed-up for 30-days. The data was collected between 21st December 2018 and 21st April 2019. Participants were followed-up for their outcome after 30 days of management ending 21st May 2019.

### Ethical considerations

Administrative permission was obtained from the Department of Surgery, Mulago National Referral and Teaching Hospital while the Makerere University College of Health Sciences-School of Medicine Research Ethics Committees (REC REF 2018-187) granted the study investigators ethical approval. Written informed consent was sought by endorsement of a pre-designed consent form either directly by the patients or by proxy through their authorized representatives. For deeply unconscious patients who could not consent or did not have legal representatives, waiver of consent was granted by the Institutional Review Board and consent was re-obtained when they regained consciousness or when their caretakers showed-up. For patients who withdrew their consent, their data were deleted. The research followed the Uganda National Council for Science & Technology (2014) guidelines on conducting research involving human subjects as participants.

### Study settings

This study was carried out at the Accident and Emergency and the Neurosurgery Departments of the Mulago National Referral Hospital located in Kampala, the capital city of Uganda (https://health.go.ug/content/mulago-national-referral-hospital). This public hospital also serves as a teaching hospital for the College of Health Sciences and School of Medicine-Makerere University Kampala (https://www.mak.ac.ug/). The hospital has a total capacity of 2,000 in-patient beds and attends to over 480,000 patients annually; including trauma patients from the busy capital city and its metropolitan area. It runs two operating theaters every day with an eight-bed general intensive care unit (ICU) and eight-bed high dependance Unit (HDU). All trauma patients are received by general and trauma surgeons who consult with a team of orthopedic surgeons, maxillofacial surgeons, physiotherapists, neurosurgeons and neurosurgery residents in a multidisciplinary approach. Because our bed occupancy rate is up to 200%, critically ill trauma patients often have to wait at the accident and emergency department until ICU or HDU space is available. The hospital has a main laboratory which conducts a wide range of investigations such as complete blood counts and clinical chemistry tests. However the consistency of bedside laboratory investigations are often a challenge due to limited supplies. Patients with TBI access a computerized tomography (CT) scanner out-of-pocket payment from a private imaging center located 1.6 km from our facility.

### Inclusion criteria

1. All adult patients admitted with severe TBI (Glasgow Comma Score (GCS) equal or less than 8) at our trauma center during the study period.
2. Patients for whom informed consent was given either directly or by proxy through their authorized representatives.

### Exclusion criteria

We excluded patients with documented conditions that could cause a spike in blood glucose or confound mortality such as:

1. Diabetes mellitus
2. HbA1c of 6.5% or more
3. Long-term steroid therapy
4. Chronic kidney disease
5. Chronic liver disease
6. Having received glucose containing fluids prior to admission at our trauma center

### Sample size calculation

Since the design was a prospective cohort study, in-part aimed at defining possible associations of study variables with 30 day mortality, it was not necessary to calculate a sample size to demonstrate a valid association with a single variable for this purpose. However owing to the possibility of differential non-response, loss to follow up and other potential factors that might affect the statistical reliability of the results, we carried out a sample size calculation. To determine the prevalence of hyperglycemia in patients with severe TBI at Mulago National Referral Hospital, we employed the Kish–Leslie formula to calculate the sample size (*N*)

(1) $$N = \displaystyle{{{{(Z\alpha )}^2}*P(1 - P)} \over {{E^2}}}$$

(2) $$Z\alpha = 1.96$$

*E* = Marginal error (0.05) for 95% confidence interval; *P* = Proportion of patients with severe TBI who had hyperglycemia. According to a study in Iran by Kafakimd et al. (2016), the proportion of patients with severe TBI who had hyperglycemia was 0.39. Substituting into the formula;

(3) $$N = \displaystyle{{{{(1.96)}^2}*(0.39)*(1 - 0.39)} \over {{{(0.05)}^2}}} = 366$$

#### Adjusting sample size for finite population

According to our electronic local hospital records, an estimated 120 patients with severe TBI had been seen in Mulago National Referral Hospital in a period of 3 months that preceded the study. Adjusting the sample size for finite population,

(4) $$Na = \displaystyle{{ne} \over {1 + \displaystyle{{ne - 1} \over n}}}$$

where *Na* is the adjusted population size, ne is the estimated sample size, and *n* is the population under study (120)

(5) $$Na = \displaystyle{{366} \over {1 + \displaystyle{{365} \over {120}}}} = 91$$

Thus a sample size of 91 participants was considered. According to a cohort study that assessed the outcome of TBI patients with acute subdural and extradural hematoma at Mulago National Referral Hospital, 9.1% were lost to follow-up during 30 days period (Ssebakumba et al., 2020). Adding 9.1% loss to follow-up to 91 yielded a final sample size of 99 participants.

### Study procedure

We measured random blood glucose (RBG) on admission and at 24 h. Venous blood was obtained and using Glucosure test kits (ISO 15197) that utilize the glucose oxidase enzyme, a reading for RBG in mmol/L was obtained. The patients were categorized based on glucose levels as hyperglycemic (RBG equal or more than 11.1 mmol/L), normoglycemic (RBG less than 11.1 mmol/L). All patients with an RBG more than 11.1 mmol/L had Hemoglobin (HbA1c) measured on the same blood sample. We captured other study variables such as demographics, mechanism of injury, concurrent illnesses, associated injuries, head and brain CT scan findings and nature of intervention on the questionnaire. Patients were subsequently followed-up for 30 days for an outcome of either dead or alive.

### Data analysis

The data were entered into EpiData software (Christiansen TB and Lauritsen JM (version 3.1)) EpiData-Comprehensive Data Management and Basic Statistical Analysis System (EpiData Association, 2010, Odense, Denmark), then exported to Stata software version 14.0 (Release 14; StataCorp 2015, College Station, TX, USA) for cleaning and analysis. Descriptive statistics for the cohort participants were summarized into means and standard deviation if the assumption of normality was fulfilled, otherwise medians and interquartile ranges were used. The proportions and percentages are presented for categorical variables. The burden of hyperglycemia was presented as a proportion. The chi-squared test was used to compare proportions of mortality between the different groups of hyperglycemia. McNemar’s test was used to compare the RBG at admission with the RBG at 24 h. To determine the relationship between hyperglycemia and mortality in severe TBI, the outcome (mortality) was dichotomized as alive or dead after 30 days of follow up. For bivariate and multivariable analysis, logistic regression was used. Atthe bivariate level only variables with *P*-values less or equal to 0.2 were considered for the multivariable modeling. Odds ratios and the 95% confidence intervals were computed. *P*-value less than 0.05 were considered to be statistically significant.

### Quality control

Data collection tools were pre-tested in similar settings. Every batch of glucometer strips was calibrated before use.

## Results

### Socio-demographic characteristics of patients with severe TBI

Most patients were male (92.7%), aged 18–30 years (47.0%) and a significant proportion reported alcohol use prior to the trauma event (36.9%) (Table 1).

**Table 1:** Showing socio-demographic characteristics of patients with severe TBI at Mulago National Referral Hospital.

Characteristic	Frequency (*n* = 99)	Percentage (%)
Sex		
Male	92	92.9
Female	7	7.1
Age		
less than 18	8	9.3
18–30	35	47
31–40	19	22.1
41–50	11	12.8
51 and above	13	15.1
Occupation		
Formal employment	5	5.1
Informal employment	61	61.6
Unemployed	12	12.1
Record missing	21	21.2
Long term Illness		
Hypertension	4	4.1
Others	73	73.7
Record missing	22	22.2
Chronic medications		
Hypertensive agents	4	4.1
Other chronic illness	73	73.7
Record missing	22	22.2
Substance abuse		
Alcohol	37	37.4
Cigarette smoking	1	1
Others	32	32.3
Record missing	29	29.3

### Prevalence of hyperglycemia in severe TBI

**Figure 1:**
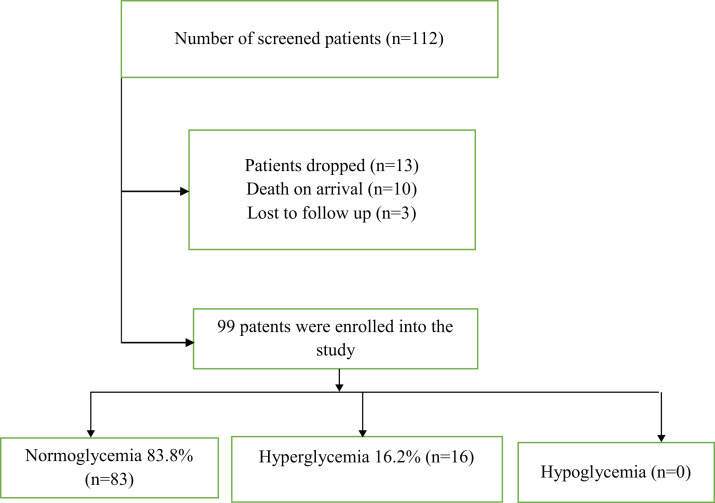
Showing prevalence of hyperglycemia in severe TBI at Mulago National Referral Hospital.

During the 4 month study period, a total of 112 patients with severe TBI were screened and out of these, 99 were enrolled into the study and completed the 30-days of follow-up. Sixteen patients were found to have hyperglycemia at admission, giving a prevalence of 16.2%. After 24 h, only 5.1% (*n* = 5) were hyperglycemic. The difference in proportions of hyperglycemic patients at admission and after 24 h was not statistically significant (McNemar test *p* = 0.083). HBA1c was done on all the patients with hyperglycemia and it was normal for all patients. None of the patients was found to be hypoglycemic (Fig. 1).

### Clinical characteristics and injuries associated with severe TBI

The most common mechanism of injury was road traffic crash (64.7%), followed by assault (17.1%) and falls from height (8.1%). Most patients presented with convulsions (48.5%) and projectile vomiting (23.2%). Limb fractures were the most common associated injury (24.7%). Many patients had contusions (33.7%), brain edema (13.3%) and epidural hematomas (10.2%) whereas a significant proportion presented with hypoxia (46.9%) (Table 2).

**Table 2:** Showing clinical characteristics and injuries associated with severe TBI at Mulago National Referral Hospital.

Factor	Characteristic	Frequency	Percentage (%)
Etiology	RTA	64	64.7
	Falls	8	8.1
	Assault	17	17.1
	Work related	1	1
History of vomiting	No	70	70.7
	Yes	23	23.2
History of convulsions	No	45	46.4
	Yes	47	48.5
	Unknown	5	5.1
Fracture	No	76	75.3
	Yes	23	24.7
Abdominal injury	No	94	95.9
	Yes	4	4.1
Facial fractures	No	90	90.9
	Yes	9	9.1
Chest injury	No	95	99
	Yes	1	1
CT Findings	Subdural hematoma	7	7.1
	Epidural hematoma	10	10.2
	Brain edema	13	13.3
	Contusion	33	33.7
	Others	17	17.3
	Missing	18	18.4
Oxygen saturation (%)	Normal > 90	52	53.1
	Hypoxia < 90	46	46.9
Systolic blood pressure	Normotensive Systolic BP > 90	74	77.1
(mmHg)			
	Hypotensive Systolic BP < 90	22	22.9
Temperature (°C)	Normothermia	66	71
	Hypothermia < 35.5	17	18.3
	Hyperthermia > 37.4	10	10.7

### Factors associated with 30-day mortality in severe TBI

**Figure 2:**
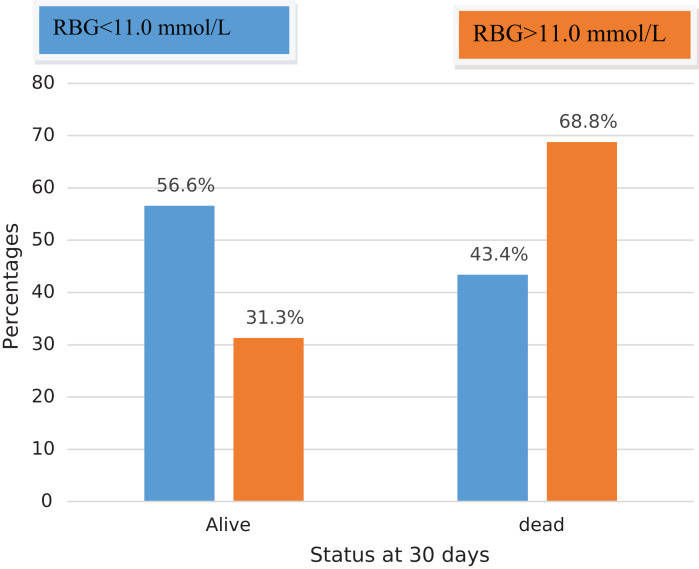
Showing mortality associated with hyperglycemia in severe TBI at Mulago National Referral Hospital.

By the end of 30 days, the overall mortality rate at 30 days was very high at 47.5% (*n* = 47) with the majority of deaths occurred in the ICU (*n* = 37, 37.4%). Only 9.1% of patients were managed operatively. The mortality rate of patients with hyperglycemia at admission was 68.8% (11/16) compared to 43.4% (36/83) in normoglycemic patients (*p* = 0.063) (Fig. 2).

In the bivariate analysis, sustaining contusions (OR 0.16; 95% CI [0.033–0.754], *P* = 0.021) and brain edema (OR 0.16; 95% CI [0.044–0.549], *P* = 0.004) were associated with low mortality (Table 3).

**Table 3:** Bi-variate analysis of variables associated with 30-day mortality in severe TBI at Mulago National Referral Hospital.

Characteristic	Alive, *n* (%)	Dead, *n* (%)	OR (95% CI)	*P*-value
RBG Status				
Normoglycemia	47 (90.4)	36 (76.6)	1	
Hyperglycemia	5 (9.6)	11 (23.4)	2.87 [0.916–9.006]	0.07
Age				
<18	5 (10.4)	3 (7.9)	1	
19–30	18 (37.5)	17 (44.7)	1.57 [0.325–7.622]	0.571
31–40	10 (20.8)	9 (23.7)	1.5 [0.276–8.138]	0.638
41–50	8 (16.7)	3 (7.9)	0.63 [0.088–4.401]	0.637
>50	7 (14.6)	6 (15.8)	1.43 [0.236–8.637]	0.698
Sex				
Male	50 (96.2)	42 (89.4)	1	
Female	2 (3.8)	5 (10.6)	2.97 [0.548–135]	0.206
Occupation				
Unemployed	3 (6.8)	2 (5.9)	1	
Formal	36 (81.8)	25 (73.5)	1.04 [0.162–6.694]	0.966
Informal	5 (11.4)	7 (20.6)	2.1 [0.251–17.594]	0.494
CT Findings				
Normal	5 (9.6)	14 (29.8)	1	
Subdural hematoma	3 (5.8)	4 (8.5)	0.48 [0.078–2.916]	0.422
Epidural hematoma	6 (11.5)	4 (8.5)	0.24 [0.046–1.210]	0.084
Brain edema	9 (17.3)	4 (8.5)	0.16 [0.033–0.754]	0.021*
Contusion	23 (44.2)	10 (21.3)	0.16 [0.044–0.549]	0.004*
Others	6 (11.5)	11 (23.4)	0.65 [0.157–724]	0.56
Oxygen saturation (%)				
normal > 90	30 (58.8)	22 (46.8)	1	
hypoxia < 90	21 (41.2)	25 (53.2)	1.62 [0.729–0.361]	0.235
Systolic BP (mmHg)				
normotensive SBP ≥ 90	39 (79.6)	35 (74.5)	1	
hypotensive SBP < 90	10 (20.4)	12 (25.5)	1.34 [0.514–476]	0.551
Temperature (°C)				
Normal temp	37 (77.1)	29 (64.4)	1	
Hypothermia < 35.5	5 (10.4)	12 (26.7)	3.06 [0.968–679]	0.057
Hyperthermia > 37.4	6 (12.5)	4 (8.8)	0.85 [0.219–3.298]	0.815
Mechanism of injury				
not recorded	4 (7.7)	5 (10.6)	1	
RTA	32 (61.5)	32 (68.1)	0.8 [0.197–254]	0.755
Falls	5 (9.6)	3 (6.4)	0.48 [0.069–3.352]	0.459
Assault	11 (21.1)	7 (14.9)	0.51 [0.101–2.574]	0.414
Substance abuse				
Alcohol intake	20 (38.5)	17 (36.2)	0.91 [0.401–2.051]	0.814
No substance abuse	33 (68.8)	26 (72.2)	1	
Other substance abuse	17 (32.7)	15 (31.9)	0.97 [0.415–2.24]	0.934
Time between injury and admission				
<1 h	15 (32.6)	11 (27.5)	1	
6 h	26 (56.5)	21 (52.5)	1.10 [0.419–898]	0.845
and above	5 (10.9)	8 (20.0)	2.18 [0.559–8.514]	0.261
Associated injuries				
Abdominal injury	3 (5.7)	1 (2.2)	0.36 [0.036–617]	0.388
Chest injuries	1 (2.0)	0 (0.0)		
Fractures, MSK (Musculoskeletal)	8 (15.4)	12 (27.3)	2.06 [0.755–5.628]	0.158
Head injury only	5 (9.6)	4 (8.5)	0.87 [0.220–469]	0.849
GCS at admission				
GCS <5 at admission	6 (13.6)	10 (23.8)	1.97 [0.648–042]	0.231
History of convulsions, yes	19 (36.5)	28 (59.6)	2.56 [1.137–5.760]	0.023

**Note:**

* *p* < 0.05 (statistically significant).

In the multi-variable analysis, having had convulsions (OR 5.49; 95% CI [1.54–19.56], *P* = 0.009) or hypothermia (OR 10.17; 95% CI [1.57–65.67], *P* = 0.015) at the time of admission was associated with high mortality. Individuals with hyperglycemia were 1.47 times likely to die at 30 days compared to those with no hyperglycemia but this association was not statistically significant (OR: 1.47; 95% CI [0.236–9.153], *P* = 0.680) (Table 4).

**Table 4:** Multivariate analysis of variables associated with 30-day mortality in severe TBI at Mulago National Referral Hospital.

Characteristic	OR (95%CI)	*P*-value
Sex		
Male	1	
Female	5.45 [0.427–69.625]	0.192
Mechanism of injury		
None	1	
RTA	2.38 [0.214–26.498]	0.48
Falls	0.66 [0.025–17.605]	0.806
Assault/work related	1.47 [0.091–23.866]	0.784
Random blood sugar		
normoglycemia	1	
Hyperglycemia	1.47 [0.236–9.153]	0.68
Temperature		
normal temp	1	
Hypothermia < 35.5	10.17 [1.574–65.669]	0.015*
hyperthermia > 37.4	2.18 [0.318–14.972]	0.427
CT Findings		
Normal	1	
subdural hematoma	1.33 [0.106–16.643]	0.824
epidural hematoma	0.11 [0.009–1.269]	0.077
brain edema	0.23 [0.023–2.281]	0.21
Contusion	0.09 [0.012–0.624]	0.015*
Others	0.71 [0.072–7.079]	0.774
Convulsions at admission		
No	1	
Yes	5.49 [1.541–19.558]	0.009*
GCS after resuscitation at admission		
5–8	1	
<5	1.51 [0.286–7.939]	0.628

**Note:**

* *p* < 0.05 (statistically significant).

## Discussion

### Socio-demographic and clinical characteristics of patients with severe TBI

In this study most patients were young adults in the productive age group of 18–30 years, comprising 47% of the study population. Males were over-represented with a male:female ratio of 9:1. This compares with similar studies done in Uganda. In a study by Ssebakumba et al. (2020), the median age affected by TBI was 29 years (IQR 23–36) with a male:female ratio of 10:1. Another study by Tran et al. (2015) found that males aged 15–29 years comprised the predominant demographic with a male:female ratio of 4.5:1. Similarly, Zia et al. (2019) studied 3,749 TBI patients, with over 70% of patients between 19–45 years age group and 80% were males. This could be linked to males being more active within occupational sectors characterized by high risk. For example, most commercial motorcycle drivers (a high risk occupation) in Uganda are male (Siya et al., 2019).

The most common cause of injury was road traffic collision (64.7%) followed by violence from assault and falls from height. These findings are similar to those of (Ssebakumba et al., 2020) who in their study of subdural and extradural hematomas at Mulago National Referral Hospital in Uganda found that 52.6% were caused by road traffic crashes. Also in a study of causes of unintentional TBIs, Zia et al. (2019) found that these were mainly due to road traffic crashes (88.9%) and falls (11.1%). Likewise, Hsia et al. (2010) documented that of the 3481 injuries studied, a majority (49%) resulted from road traffic crashes. These findings are also similar to those done within the East African region for example in Tanzania (Chalya et al., 2012) and in other countries such as Malaysia (91.5%) (Haron, Musa & Haspani, 2011). In our settings, this pattern of injury is mainly due to the fact that majority of youths ride motorcycles as a source of income and are thus at high risk of road traffic crashes, particularly head injuries due to the non-use of protective helmets (Chalya et al., 2014). For instance in Uganda, only 1% of passengers on motorcycles wear helmets (Roehler et al., 2013), partly due to weaker legislation and law enforcement. Also, motorcycles are often involved in theft cases and hence motorcycle drivers are victims of assault from mob-violence (Doherty, 2017).

Over 48% of our patients presented with convulsions associated with projectile vomiting (70%), hypothermia (18%), hypotension (23%) and hypoxia (47%) respectively. Although these could contribute to the trauma death triad resulting from secondary brain ischemic insults, we were not able to do invasive intracranial pressure monitoring in our low resource setting but rather to provide the minimum affordable health care package of pulse-oximetry for oxygen circulation, fluids to correct hypotension, mannitol for cerebral edema, anti-convulsants and oxygenation. These measures had been proven to improve TBI outcomes in our setting (Ssebakumba et al., 2020).

Other associated injuries in severe TBI were; limb fractures (17.8%), chest injuries (4.8%) and abdominal injuries 4.8%; indicating poly-trauma which emphasized the need for a multi-disciplinary trauma team. The most common brain CT scan finding was that of contusions in (33.7%), followed by cerebral edema (13.3%). These along with diffuse axonal injuries being amenable to medical treatment to some extent, could partly explain why only 9% of our study population underwent a surgical intervention as would be the case for extra-axial hematomas whose main treatment is surgical decompression (Ssebakumba et al., 2020).

However, the fact that we have limited surgical theaters and intensive care space that limits access to surgery and a prognostication of high mortality in severe TBI before optimization for surgery; cannot be excluded as an account for low rates of surgical intervention as opposed to conservative management for these patients. It is also important to note that about 18.4% of our patients with severe TBI did not have a brain CT scan because they could not afford the expensive investigation done at a cost of 70 USD, thus the anatomical lesions in our study could be under-reported. In addition, with limited diagnostics its challenging for the neurosurgical team to intervene for such patients, warranting the need for improved quality of trauma care in low-income countries.

### Prevalence of hyperglycemia in severe TBI

One of the study aims of the present study was to determine the prevalence of hyperglycemia resulting from the trauma-related stress response in severe TBI. We found that 16.2% (1 in 6 patients) admitted with severe TBI had hyperglycemia. Despite the same case definition for hyperglycemia (RBG equal or more than 11.1 mmol/L), our findings contrast with those in Nigeria by Adeolu et al. (2015), who found that only 2.5% (1 in 37) patients with TBI had hyperglycemia. Although Adeolu et al. (2015) attributed their findings to possible absence of hyperglycemic response in TBI patients in the African population, despite them including mild, moderate and severe TBI. The results from our stratified inclusion of only severe TBI patients suggest that the hyperglycemic stress response to trauma in TBI patients indeed happens in the African population.

Kumar et al. (2015) found that blood glucose levels were significantly higher in patients with severe TBI compared to those with mild and moderate TBI at admission. In fact in an earlier study by Adeolu et al. (2010), the only patient who was documented to have hyperglycemia had severe TBI. Our findings are comparable to those of (Kafakimd et al., 2016) who documented that 39% (85/220) of patients with severe TBI had hyperglycemia and are in conformity with those published by Bosarge et al. (2015), who found that 24.3% (152/626) had stress induced hyperglycemia. This finding highlights the fact that hyperglycemic stress response in severe TBI can occur any population.

### Factors associated with 30-day mortality in patients with severe TBI

First, we found that the mortality rate of severe TBI patients with hyperglycemia was 68.8% (OR = 1.47; *P* = 0.064) compared to (43.4%) in those without hyperglycemia. Our findings are incongruent with those from a Nigerian study that found no mortality in the 4 patients with hyperglycemia out of 146 TBI patients studied (Adeolu et al., 2015). According to Salehpour et al. (2016) in their prospective study of 80 consecutive patients with severe TBI, of the 25% in-hospital mortality reported, the mean RBG was comparable between the dead and discharged patients (*P* = 0.91) (Salehpour et al., 2016). In another study by Adeolu et al. (2010), out of the 50% mortality registered, none of the patients had hyperglycemia. In the latter, the authors suggested that the critical level for hyperglycemia in severe TBI in the African population may be lower compared to other parts of the world.

However, our findings suggest that this may not necessarily be the case. In addition to the (11/16) mortality in the hyperglycemic group, we found that all 5 patients who had hyperglycemia at 24 h, had been hyperglycemic at admission but with normal ranges of HbA1c. Besides, the number of hyperglycemic individuals dropped to 5 at 24 h due to mortality of those who were admitted with hyperglycemia. Though not statistically significant, our findings are similar to those established by Kafakimd et al. (2016), who documented the mortality rate in severe TBI patients with hyperglycemia of 65.8% (*n* = 56) against 23.7% (*n* = 32) within the group that had normal blood glucose, demonstrating that mortality increased as blood glucose level increased (chi2 = 5.033; *P* = 0.014). Whereas their study was conducted in an entirely different population in the Middle East, our findings show that similar pathophysiological processes occurring following trauma could account for the presence of hyperglycemia in severe TBI and its effect on patient outcomes.

Similarly in a study of 834 severe TBI patients, Salim et al. (2009) found that persistent hyperglycemia was associated with a five-fold increase in mortality. These findings were consistent with those of Bosarge et al. (2015), who noticed a 50% increase in mortality amongst hyperglycemic severe TBI patients compared to those with normal blood sugar levels. Even though these results partly compare with those from our study, there was no statistically significant association between hyperglycemia and mortality in severe TBI patients in our cohort. The discrepancy could be attributed to the difference in follow-up period and to the small sample size in our study compared to that of Kafakimd et al. (2016) and Salim et al. (2009) of 220 and 834 patients respectively.

In addition, we found that the presence of convulsions was a statistically significant predictor of mortality by 5.46 fold (*P* = 0.009) and this was compared to an 8.9 fold risk of 30-day mortality documented in patients with symptomatic seizures by Hesdorffer et al. (2009). Thus control of convulsion is a well-documented neuro-protective strategy in averting mortality (Wells & Hutchinson, 2018) and morbidity due to TBI (Desai & Jain, 2018).

Also, hypothermia below 35.5 °C increased the risk of 30-mortality by 10 fold (*P* = 0.015). Although hypothermia is physiologically presumed to be protective to the injured brain, this topic remains controversial as validation clinical trials and systematic reviews have failed to demonstrate high quality evidence on its efficacy, in terms of reducing mortality and adverse outcome of TBI patients (Ahmed, Bullock & Dietrich, 2016; Lewis et al., 2017). The neuro-protective effects of hypothermia are theoretically able to decrease the tissue metabolic rate and in doing so restore the oxygen supply, limiting excitotoxicity thus reducing ATP depletion and slowing free radical production which prevents cell death (Andresen et al., 2015). However, hypothermia and the cooling methods can be associated with risks of pneumonia, coagulopathy, electrolyte imbalance and cardiac arrhythmias, depending on temperature protocols used (Urbano & Oddo, 2012). In addition, the shivering arising from hypothermia could increase the brain consumption energy and intracranial pressure which is one of the important determinants of TBI outcome (Urbano & Oddo, 2012). However in our study, the increased mortality associated with hypothermia at admission could have resulted from prolonged hypovolemic shock and overexposure prior to hospital arrival as we do not have a formal pre-hospital care system in our setting. In fact, the majority of our patients arrive by public taxis. Such hypothermia is known to contribute to the death triad (Keane, 2016), as opposed to the therapeutically induced hypothermia rightly termed as target temperature management, executed under well-established rewarming protocols among physiologically normothermic patients in intensive care units (Nunnally et al., 2011). Besides, the ultimate objective of therapeutic hypothermia would be to preserve the central nervous system, while buying time to resolve the primary lesion (Andresen et al., 2015), for which case our study population, restricted to severe TBI with anticipated prolonged brain damage is not the ideal to end this argument.

Our study found that sustaining a contusion was associated with lower odds for mortality (OR = 0.09, *P* = 0.015). These findings agree with a study by Adatia, Newcombe & Menon (2020), in which mortality due to brain contusions has been linked to the ability to predict their clinical or radiological deterioration before progression. Using lessons learned from a study on determinants of outcome of extra-axial hematoma (Ssebakumba et al., 2020), it was possible for the attending clinicians in this study to predict which patients with contusions would deteriorate by risk stratification based on their smoking status, admission GCS, systolic BP, initial contusion size and location and co-existence of extra-axial hematoma and cerebral edema on brain CT image, to discern who needed an emergency decompressive craniectomy. In contrast some researchers have attributed higher mortality in brain contusions to increasing age (Kirkman et al., 2013) and our study generally involved a younger population. Our findings showed no statistically significant association between hypotension, hypoxia, mechanism of injury and injuries to other systems with 30-day mortality. These variables are however key to preventing secondary brain insults and in predicting a favorable outcome in terms of morbidity (Ssebakumba et al., 2020).

### Study limitations

This study was not without limitations. First, the study was approved for a brief period of 4 months and an additional 1 month of follow-up which highlights a relatively small consecutive sample that could limit the generalizability of results. Secondly, our findings are based on 24-h admission, socio-demographic and clinical characteristics without considering other in-hospital management and complications such electrolyte imbalance, serum glucose level management and hospital acquired infections that could influence the outcome of TBI. In addition, the study was limited to only patients with severe TBI, thus the pattern of blood glucose in all patients with TBI remains largely unknown in our setting. Finally, although the study was conducted in the country’s top referral hospital where very critically ill patients are expected and thus mortality, a good number of patients died on arrival and many such patients fail to reach the emergency department due to lack of a formal pre-hospital care in our setting. Thus our mortality rate and so the prevalence of hyperglycemia could be under-reported as they don’t represent such missed cases.

## Conclusion

Early hyperglycemia following severe TBI does occur in (16.2%) with a tendency towards a high mortality rate. Hypothermia and convulsions at admission were major predictors of mortality in severe TBI. These findings justify routine serum glucose monitoring in severe TBI and could form a basis for establishing a management protocol for such patients in resource constrained settings. Future studies should be multi-center randomized controlled trials with diverse population to provide meaningful conclusive deductions on association between hyperglycemia and mortality in severe TBI.

## Supplemental Information

10.7717/peerj.10589/supp-1 Supplemental Information 1 Dataset.

10.7717/peerj.10589/supp-2 Supplemental Information 2 Questionnaire.

## Additional Information and Declarations

### Data Availability

The following information was supplied regarding data availability: The raw data are available in the Supplemental Files.
