# Supplementary material for: Hyperglycemia in severe traumatic brain injury patients and its association with thirty-day mortality: a prospective observational cohort study in Uganda
Source: PeerJ. 2021 Jan 15;9:e10589. doi: 10.7717/peerj.10589 (PMC7812933; doi:10.7717/peerj.10589)
Supplement: Supplemental Information 2 [file peerj-09-10589-s002.pdf]

APPENDIX IV: QUESTIONNAIRE

Hyperglycemia in severe Traumatic Brain Injury and its association with thirty-day mortality in Mulago National Referral Hospital (MNRH), Uganda

Fill in the available spaces or tick appropriately

Code NO.		Date		
1. Age :……….(in yrs.)		2.	Sex: a)Male	b) Female
3. Address:……………………		4.	Occupation (specify):	
Phone No.1	Phone No.3	……………………………….		
Phone No.2				
**5.Marital status**		**6. Cause of TBI**		
a)Single		a) RTA		
b)Married		b) Falls		
c)Widowed		c) Assault		
d)Divorced		d) Domestic violence		
		e) Work related		
**7. Who brought the patient to Hospital?**		**8. Mode of arrival**		

a) Police		a) Ambulance		b) Police Pick-up
b) Nurse from referral site		c) Private vehicle		d) Public vehicle
c) Well-wishers		e) Motorcycle		f) Bicycle
d) Attendants		g) Other(Specify)		
e) Other(Specify)				
**9. Time between injury and arrival to**		**10. Pre-hospital treatment**		
**Hospital**		a. Oxygen		b. IV fluids

a. <1hr	b. 1-6hrs	c. Mannitol	d. Antibiotics
c. 6- 12hrs	d. 12-24hr	e. Analgesia	f. Intubation
e. 24-48hrs	f. >48hrs	g. Steroids	h. Glucose
**11.Long term illness**			
a. Diabetes Mellitus	b. Hypertension	c. Chronic Renal Disease	d. Chronic disease liver
e. Others			
**12. Chronic medication**			
a. Steroid therapy	b. Hypoglycemia agents	c. Antihypertensive	d. others
**13.Substance abuse**			
a. Alcohol	b. Cigarette smoking	c. Cocaine	d. Others

i. Other (specify)………………………………………

**11.Vomiting:Yes No**			12.	Convulsions: Yes No
**13.Post-resuscitation GCS**	**Total**		**14.**	**Pupillary response and size**
a. Eye opening				a. Normal
a. Verbal Response<br>b. Motor response				b. Dilated right pupil
Systolic BP (mmHg) <90	>90			
Pulse Rate/min				c. Dilated left pupil
Respiratory Rate /min<br>Temperature (°C)<br>Oxygen saturation(SpO_2) <90%		>90%		

**15.RBSmmol/L**<br>a. At Admission<br>b. After 24hrs		**16. HBA1c**
**17. CSF Leak**		**18. Limb weakness**
a)Nose Both R/L		a)Upper limbs RL
b)Ear Both R/L		b)Lower limbs RL
19.**Peri-orbital ecchymosis** Yes	No	22. **Fractures** (Specify the site)
20.**Abdominal injury**(specify)		23. **Facial fracture**
21.**Chest injury**(specify)		24. **Neck stiffness** Yes No
**25.Laboratory parameters**		**26. Time between admission and surgery**
a. Hemoglobin (Hb)		a) <6hrs
b. Serum sodium		b) 6-24hrs
c. Serum potassium		c) 24-48hrs
**27.Cause of delay**		**28. Findings at surgery**
a) Lack of supplies<br>b) Lack of theatre space		a) Subdural hematoma<br>b)Epidural hematoma<br>c) Brain edema<br>d) Contusion<br>e) Laceration
c) Later development of features of raised		
ICP		f) Other(Specify

**29. Surgery done**<br><br>a) Decompressive craniectomy<br><br>b)Osteoplastic decompressive craniotomy<br>c) Duroplasty	**30. Other surgical intervention**<br><br>i)Tracheostomy<br><br>ii)Thoracostomy<br>iii)Laparotomyiv)Fracture management<br>**v)**Other (Specify)
**31. ICU**<br><br>Number of days on ventilation<br><br>GCS at discharge from ICU<br><br>a.Motor response<br>b.Eye opening<br><br>c.Verbal response<br>Death in ICU	**32. Laboratory parameters in ICU**<br><br>a. Hemoglobin (Hb)<br><br>b. Glucose (mmol/L)<br><br>c. Serum sodium<br>d. Serum potassium<br><br>e. Urea<br>f. Creatinine
**33. Emergency/Ward 1**<br><br>a. Wound infection<br>b. Extradural CSF leak<br>c. Meningitis<br>d. Swelling at the operation site<br>e. Abscess	**34.status at 30 days**<br><br>a. Alive<br>b. Dead

APPENDIX III: GLASGOW COMA SCALE

Parameter	Response	Score
Eye opening	None	1
	To pain	2
	To speech	3
	spontaneously	4
Verbal Response	None	1
	Incomprehensible sounds	2
	Inappropriate words	3
	Confused	4
	Well Oriented	5
Motor Response	None	1
	Abnormal extension	2
	Abnormal Flexion	3
	Withdraws from pain(normal flexion)	4
	Localizes pain	5
	Obeys commands	6
